# Sex differences in auditory fear discrimination are associated with altered medial prefrontal cortex function

**DOI:** 10.1038/s41598-020-63405-w

**Published:** 2020-04-14

**Authors:** Harriet L. L. Day, Sopapun Suwansawang, David M. Halliday, Carl W. Stevenson

**Affiliations:** 1 School of Biosciences, University of Nottingham, Sutton Bonington Campus, Loughborough, LE12 5RD UK; 2 Department of Electronic Engineering, University of York, Heslington York, YO10 5DD UK; 3 Present Address: RenaSci Ltd, BioCity, Pennyfoot Street, Nottingham, NG1 1GF UK; 4 Present Address: Faculty of Science and Technology, Nakhon Pathom Rajabhat University, 85 Malaiman Road, Muang, Nakhon Pathom 73000 Thailand

**Keywords:** Fear conditioning, Neural circuits

## Abstract

The increased prevalence of post-traumatic stress disorder (PTSD) that is observed in women may involve sex differences in learned fear inhibition and medial prefrontal cortex (mPFC) function. PTSD is characterized by fear overgeneralization involving impaired fear regulation by safety signals. We recently found that males show fear discrimination and females show fear generalization involving reduced safety signalling after extended fear discrimination training. Here we determined if these sex differences involve altered mPFC function. Male and female rats underwent three days of auditory fear discrimination training, where one tone (CS+) was paired with footshock and another tone (CS−) was presented alone. Local field potentials were recorded from prelimbic (PL) and infralimbic (IL) mPFC during retrieval. We found that males discriminated and females generalized based on cue-induced freezing at retrieval. This was accompanied by sex differences in basal theta and gamma oscillations in PL and IL. Importantly, males also showed PL/IL theta activation during safety signalling by the CS− and IL gamma activation in response to the threat-related CS+, both of which were absent in females. These results add to growing evidence indicating that sex differences in learned fear inhibition are associated with altered mPFC function.

## Introduction

The prevalence of anxiety and trauma-related disorders (e.g. post-traumatic stress disorder (PTSD)) is much greater in women than in men^1,2^. Growing evidence points to sex differences in learned fear inhibition and the neural circuitry underlying its regulation as key players in this increased disease prevalence in women^3^. One form of learned fear inhibition impaired in PTSD is fear extinction, which is the decrease in fear resulting from repeated or prolonged non-reinforced presentation of the conditioned cue or context. Medial prefrontal cortex (mPFC) is crucial for learned fear and its inhibition through extinction^4^, while PTSD is associated with mPFC dysfunction^5,6^. Recent studies have demonstrated sex differences in fear extinction involving mPFC function^7–9^. Oscillatory activity is important for various prefrontal-dependent cognitive and memory processes, in part by mediating functional interactions between mPFC and other inter-connected areas involved in coordinating these processes^10,11^. This includes fear extinction, which involves theta and gamma oscillations in mPFC^12,13^. We have shown in rats that enhanced learned fear expression and reduced extinction recall in females, compared to males, are accompanied by sex differences in these prefrontal oscillations^14,15^.

In contrast to fear extinction, little is known about the neural mechanisms underlying sex differences in other types of learned fear inhibition. During fear discrimination, one conditioned stimulus (CS+) predicts threat by becoming associated with an aversive unconditioned stimulus (US; e.g. footshock), while another conditioned stimulus (CS−) signals safety by predicting the non-occurrence of the US^16^. Fear discrimination is thought to be a form of learned fear inhibition by the CS− and the overgeneralization of fear to innocuous cues, which is a feature of PTSD, has been conceptualized as a deficit in fear regulation involving impaired safety signalling^17^. Recent studies have shown sex differences in cued and contextual fear discrimination^18–25^. We recently showed in rats that males display auditory fear discrimination after extended discrimination training, whereas females exhibit fear generalization involving reduced safety signalling^26^. However, the neural basis of sex differences in cued fear discrimination remains unclear.

In this study we determined if sex differences in auditory fear discrimination involve altered mPFC function. As with fear extinction, mPFC plays a key role in fear discrimination^27–33^. Moreover, theta and gamma oscillations in mPFC are also implicated in fear discrimination^34,35^. After extended auditory fear discrimination training, we recorded local field potentials (LFPs) in mPFC during retrieval testing to determine if discrimination and generalization in males and females, respectively, are characterized by sex differences in mPFC function. The prelimbic (PL) and infralimbic (IL) subregions of mPFC play opposing roles in the expression and extinction of learned fear^4^ and we have previously shown sex differences in theta (4–12 Hz) and low gamma (30–45 Hz) oscillations in these areas during learned fear expression and extinction^14,15^. As there is also evidence for distinct contributions of PL and IL to fear discrimination^29,36^, we recorded LFPs from both of these mPFC subregions at retrieval.

## Results

### Males discriminate and females generalize at retrieval after extended fear discrimination training

**Figure 1:**
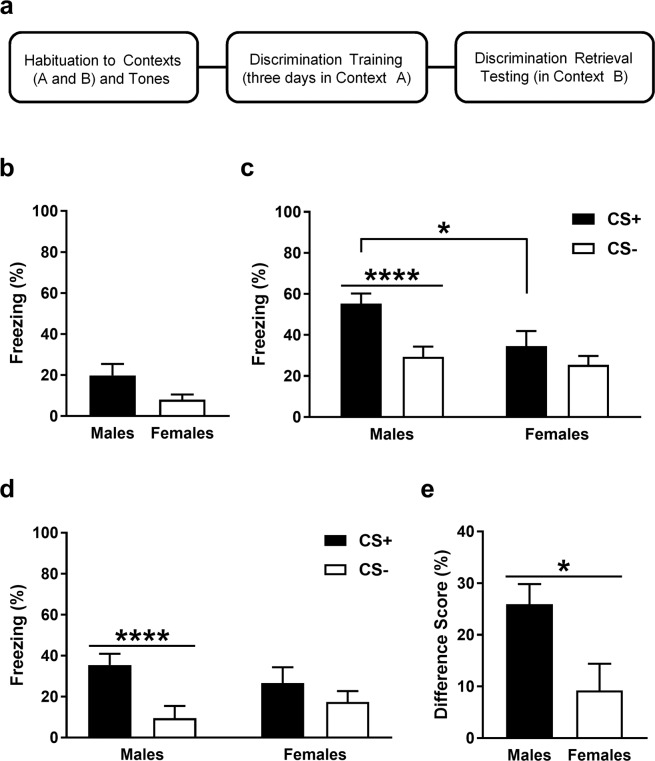
After extended auditory fear discrimination training, males show fear discrimination and females show fear generalization at retrieval. (**a**) Schematic representation of the fear discrimination paradigm used. (**b**) Baseline freezing before cue presentations at retrieval. There was no difference in freezing between males and females. (**c**) Freezing during the CS+ and CS− at retrieval. Freezing was increased during the CS+ in males, compared to females (*P < 0.05). Freezing was also increased during the CS+, compared to the CS−, in males (****P < 0.0001) but not in females. (**d**) Freezing during the CS+ and CS− at retrieval, with baseline freezing subtracted. Freezing was increased during the CS+, compared to the CS−, in males (****P < 0.0001) but not in females. (**e**) Difference score indicating the difference in freezing between the CS+ and CS−. Males showed a greater difference score, compared to females (*P < 0.05).

The auditory fear discrimination paradigm used is depicted in Fig. 1a and baseline freezing before cue presentations during retrieval is shown in Fig. 1b. Males (n = 19) showed more freezing than females (n = 13) but an unpaired t-test revealed that this difference was not significant (t_30_ = 1.65, P = 0.11). This suggests that there were no sex differences in contextual fear before cue presentations at retrieval. Freezing in response to the CS+ and CS− at retrieval is shown in Fig. 1c. Two-way analysis of variance (ANOVA) revealed a significant sex x CS interaction (F _(1, 30)_ = 6.8, P = 0.014). Post-hoc analysis indicated that freezing during CS+ presentation was significantly greater in males, compared to females (P < 0.05). Males also showed significantly increased freezing during the CS+, compared to the CS− (P < 0.0001), whereas females showed no difference in freezing between the CS+ and CS− (P > 0.05). These results indicate that males show fear discrimination based on cue-induced freezing at retrieval after three days of discrimination training. They also suggest that females show reduced fear expression and/or discrimination, compared to males. Because there were qualitative differences between males and females in baseline freezing before cue presentations at retrieval, we also examined freezing in response to the CS+ and CS− with baseline freezing subtracted^23^, which is shown in Fig. 1d. Two-way ANOVA revealed a significant sex x CS interaction (F _(1, 30)_ = 6.8, P = 0.014). Post-hoc analysis indicated that males showed significantly increased freezing during the CS+, compared to the CS− (P < 0.0001), while females showed no difference in freezing between the two cues (P > 0.05). Importantly, males and females did not differ in freezing during CS+ presentation (P > 0.05), suggesting the lack of a sex difference in fear expression. We also calculated a difference score by subtracting freezing in response to the CS− from freezing in response to the CS+^34^, which is shown in Fig. 1e. An unpaired t-test revealed that the difference score was significantly greater in males, compared to females (t_30_ = 2.61, P = 0.014). Taken together, these results suggest that females show fear generalization after three days of discrimination training.

### Males and females show differences in basal theta and gamma activity in mPFC at retrieval

**Figure 2:**
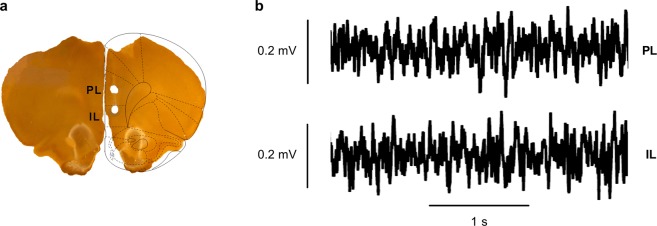
Histological verification of electrode placements in and sample LFP traces recorded from PL and IL at retrieval. (**a**) Representative example of electrode placements as indicated by lesions in PL and IL (superimposed schematic is adapted from^58^). (**b**) Examples of LFP traces recorded from PL (top) and IL (bottom) before cue presentations during retrieval (after high-pass filtering at 4 Hz).

**Figure 3:**
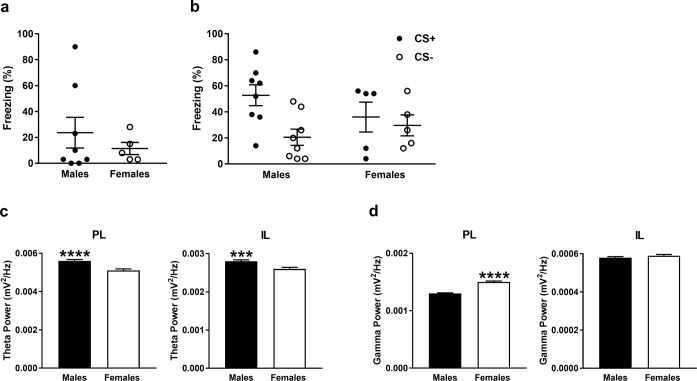
**(a**,**b**) Freezing at retrieval in the males and females from which LFP data in mPFC was obtained and analyzed. (**a**) Baseline freezing before cue presentations during retrieval. (**b**) Freezing during the CS+ and CS− at retrieval. (**c**,**d**) Basal LFP activity in PL and IL before cue presentations at retrieval in males and females. (**c**) Males showed significantly greater basal theta power in PL (****P < 0.0001) and IL (***P < 0.001), compared to females. (**d**) Females showed significantly greater basal gamma power in PL (****P < 0.0001), but not in IL, compared to males.

An example of electrode placements in PL and IL is shown in Fig. 2a and examples of LFPs recorded from PL and IL before cue presentations at retrieval and then high-pass filtered at 4 Hz are shown in Fig. 2b. Only rats with confirmed electrode placements in both PL and IL were included in the LFP data analysis, although some of this data needed to be omitted from the analysis because it contained electrical noise artefacts. The final dataset included n = 8 males and n = 5 females (the behavioural data for these animals alone is shown in Fig. 3a,b). Basal theta (4–12 Hz) power in PL and IL before cue presentations at retrieval in males and females is shown in Fig. 3c. Males showed significantly greater basal theta power in PL (log_10_ (F_11590, 7300_) = 0.039; P < 0.0001) and IL (log_10_ (F_11590, 7300_) = 0.033; P = 0.00015), compared to females. Basal gamma (30–45 Hz) power in PL and IL before cue presentations at retrieval in males and females is shown in Fig. 3d. Females showed significantly increased basal gamma power in PL (log_10_ (F_25498, 16060_) = −0.065; P < 0.0001), but not IL (log_10_ (F_25498, 16060_) = −0.0091; P = 0.07), compared to males. These results indicate sex differences in basal theta and gamma activity in mPFC before cue presentation at retrieval.

### Males, but not females, show theta activation in mPFC during safety signalling by the CS−

**Figure 4:**
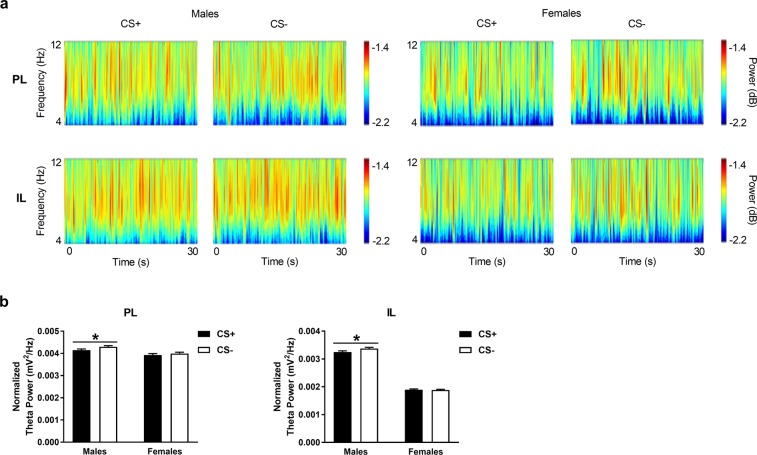
Theta power in PL and IL during CS+ and CS− presentation during retrieval in males and females. (**A**) Wavelet periodograms showing theta (4–12 Hz) power, which is represented by different colors in the adjacent scale bars (dark blue: low power, red: high power), over time during the CS+ and CS−. No obvious qualitative differences in theta power were apparent between the CS+ and CS− in PL (top) or IL (bottom) in males (left) or females (right). (**B**) In males, theta power averaged over the frequency range and cue duration was significantly increased in response to the CS−, compared to the CS+, in PL and IL (*P < 0.05), whereas there were no differences in theta power between the two cues in females.

Theta power in PL and IL in response to the CS+ and CS− during retrieval in males and females is shown in Fig. 4. We first examined qualitative differences in theta power throughout the frequency band (4–12 Hz) and duration of the cues (30 sec each) via visual inspection of the wavelet periodograms (Fig. 4a). No obvious differences in theta power were observed during CS+ vs CS− presentation in either area in males or females. We then examined quantitative differences in theta power averaged over the frequency band and cue duration using multi-taper analysis (Fig. 4b). Theta power during cue presentations was normalized to basal theta power given our finding of sex differences in basal theta power in mPFC. In males we found a significant increase in theta power in response to the CS−, compared to the CS+, in PL (log_10_ (*F*_*13680, 12600*_) = −0.015; P = 0.023) and IL (log_10_ (*F*_*13680, 12600*_) = −0.016; P = 0.018). However, in females we found no such difference in theta power between CS+ and CS− presentation in PL (log_10_ (*F*_*8280, 8640*_) = −0.007; P = 0.22) or IL (log_10_ (*F*_*828, 864*_) = 0.002; P = 0.39). These results suggest that males show theta activation in mPFC during safety signalling by the CS−, which is absent in females.

### Males, but not females, show IL gamma activation in response to the threat-related CS+

**Figure 5:**
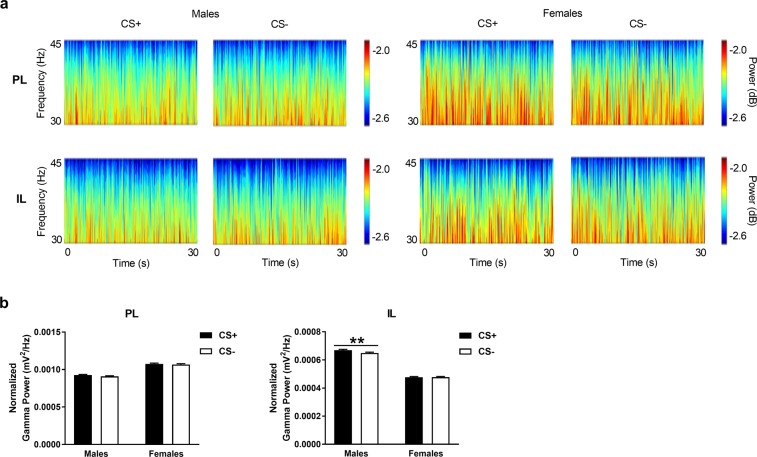
Gamma power in PL and IL during CS+ and CS− presentation at retrieval in males and females. (**A**) Wavelet periodograms showing gamma (30–45 Hz) power, which is represented by different colors in the adjacent scale bars (dark blue: low power, red: high power), over time during the CS+ and CS−. No obvious qualitative differences in gamma power were observed between the CS+ and CS− in PL (top) or IL (bottom) in males (left) or females (right). In males, gamma power averaged over the frequency range and cue duration was significantly increased in response to the CS+, compared to the CS−, in IL (**P < 0.01) but not in PL. In contrast, females showed no differences in gamma power between the two cues.

Gamma power in PL and IL during CS+ and CS− presentation at retrieval in males and females is shown in Fig. 5. Again, we initially examined qualitative differences in gamma power throughout the frequency band (30–45 Hz) and duration of the cues (30 sec each) by visually inspecting the wavelet periodograms (Fig. 5a). As was the case for theta power, we observed no obvious differences in gamma power during CS+ vs CS− presentation in PL or IL in either sex. We next used multi-taper analysis to examine quantitative differences in gamma power averaged over the frequency band and cue duration (Fig. 5b). Gamma power during cue presentations was normalized to basal gamma power given our finding of sex differences in basal gamma power in mPFC. We found that males showed a significant increase in gamma power in response to the CS+, compared to the CS−, in IL (log_10_ (*F*_*30096, 27720*_) = 0.013; P = 0.0053) but not in PL (log_10_ (*F*_*30096, 27720*_) = 0.007; P = 0.056). However, we found no difference in gamma power between CS+ and CS− presentation in PL (log_10_ (*F*_*18126, 19008*_) = 0.0034; P = 0.30) or IL (log_10_ (*F*_*18216, 19008*_) = −0.0008; P = 0.45) in females. These results suggest that males show IL gamma activation in response to the threat-related CS+, which, again, is absent in females.

## Discussion

In this study we sought to determine if sex differences in auditory fear discrimination involve altered mPFC function. After three days of discrimination training we found that males show fear discrimination and females show fear generalization at retrieval, which were associated with sex differences in mPFC oscillations. While males showed much greater basal theta power in PL and IL, females showed much greater basal gamma power in PL, but not IL, before cue presentations at retrieval. In response to cue presentations at retrieval, males showed increased theta power in PL and IL during the CS−, compared to the CS+, while IL gamma power was increased during the CS+, compared to the CS−. Notably, females showed no such differences in mPFC activity between the CS+ and CS−. Taken together, these results suggest that sex differences in fear discrimination after extended training may involve altered basal oscillatory activity in mPFC. They also raise the possibility that fear discrimination in males involves mPFC theta activation during safety signalling by the CS− to suppress learned fear expression and IL gamma activation in response to threat when discriminating between the CS+ and CS−. In contrast, fear generalization in females may result from reduced safety signalling by the CS− due to a lack of mPFC theta activation and reduced discrimination between the CS+ and CS− due to a lack of IL gamma activation.

Our finding that males and females discriminated and generalized, respectively, at retrieval after three days of discrimination training replicates our previous results^26^ and broadly agrees with other evidence of sex differences in fear discrimination. Previous studies have shown in males that more training results in improved fear discrimination^16,37,38^. Recent studies have also shown reduced fear discrimination (i.e. more fear generalization) in females, compared to males^18–22,24,25^. In the present study it is worth noting that, compared to males, the lack of difference in freezing during CS+ vs CS− presentation in females was driven largely by a relative decrease in freezing to the CS+ and not an increase in freezing to the CS−. Thus one could argue that our results may simply reflect reduced fear expression rather than fear generalization in females. However, when baseline freezing before cue presentations was accounted for in freezing levels during CS+ and CS− presentation then a sex difference in fear expression was no longer observed. We also previously found evidence of reduced safety signalling by the CS− in females^26^. This was demonstrated in a retardation test^17^, where the CS− was used as the cue in later fear conditioning, and males, but not females, showed reduced (or retarded) learning of the cue-shock association. Although not examined here, this lack of safety signalling in females suggests that the present results are better explained by fear generalization than by reduced fear expression. It is also possible that females instead displayed lower freezing levels because they were more active generally or expressed more active fear responding than males. We found previously that females display increased locomotor activity, compared to males^26^. Females have also been reported to display more active darting behaviour as a learned fear response than males^25,39,40^, although other studies have found little evidence of darting in females^23,41^. We are characterizing darting during fear discrimination and its retrieval in our ongoing research to address this important issue.

In contrast to our results, other studies have found no sex differences in fear discrimination^42–45^. Another study found enhanced cued fear discrimination in females, compared to males, with one training session but no sex differences in fear discrimination or safety signalling with repeated training sessions^23^. The latter was assessed using a summation test, where presentation of the CS+ and CS− together reduces fear, compared to CS+ presentation alone, if the CS− signals safety^17^. It is unclear why their results differed from ours but this was likely due to methodological differences between the studies. That study used auditory or visual cues for the CS+ and CS−, whereas we used two auditory cues. Therefore these discrepant findings could be due, at least in part, to differences in discriminating between cues from the same or different sensory modalities. It is also possible that the discrepancies in safety signalling as inferred from retardation and summation testing involve differences in the psychological and/or neural mechanisms underpinning these two processes^46^.

Our finding of fear discrimination in males accompanied by differences in oscillatory activity in mPFC during CS+ vs CS− presentation at retrieval adds to growing evidence of the involvement of this region in fear discrimination^27–36^. Although we found quantitative differences in theta and gamma power between CS+ and CS− presentation when activity was averaged over the entire frequency band and cue duration (see below), no qualitative differences were apparent when activity was examined throughout each frequency band or cue duration. This lack of qualitative differences in LFP power between the CS+ and CS− was most likely due to each animal expressing freezing at different times during the repeated cue presentations. Previous studies examining the individual roles of PL and IL in fear discrimination have found mixed results. While there is evidence indicating that both of these mPFC subregions are involved^32^, other studies have demonstrated distinct contributions of PL and IL to fear discrimination^29,36^, which is mirrored in the present study. We found IL gamma activation in response to the CS+ during retrieval, in keeping with other evidence indicating that this area is involved in fear discrimination^29,36^. Gamma oscillations mediate the functional connectivity between mPFC and primary auditory cortex that underpins successful auditory fear discrimination^35^, highlighting their importance for mediating communication within the neural circuitry underlying fear discrimination. Interestingly, we found that fear generalization in females was associated with a lack of gamma activation in IL at retrieval. We have shown previously that extinction retrieval in males was accompanied by IL gamma activation, whereas females showed reduced extinction retrieval associated with a lack of gamma activation in IL^15^. In the present study we also found much greater basal gamma activity selectively in PL in females, compared to males, before cue presentations at retrieval. Evidence indicates that functional coupling between PL and IL plays a role in different fear memory processes, therefore it is possible that this enhanced basal gamma activity in PL also contributes to fear generalization in females by modulating cue-induced gamma activation in IL^4^. Taken together, these findings suggest that mPFC gamma oscillations are involved in sex differences in learned fear inhibition more generally.

In contrast to gamma oscillations, we found that males showed theta activation in both PL and IL in response to the CS− during fear discrimination retrieval. At first glance these results seem to contradict our earlier findings showing theta activation in PL, but not IL, during learned fear expression^14^. However, in that study a single CS was used, suggesting that theta oscillations might play different roles in fear expression and discrimination. PL theta activation might be sufficient for learned fear expression but more complex tasks like fear discrimination may require theta activity in both PL and IL^4,32^. Another possibility is that volume conduction of theta oscillations occurred between PL and IL. Evidence indicates that prefrontal theta reflects local activity and not activity that is volume conducted from other distant areas^47^. Moreover, we showed previously that learned fear expression is associated with PL, but not IL, theta activation^14^. Nevertheless, volume conduction of theta oscillations may have occurred between these adjacent mPFC subregions. A previous study showed mPFC theta activation during CS+ presentation at retrieval with successful auditory fear discrimination^34^, which, again, would appear to be at odds with our results. However, methodological differences between the studies may account for this apparent discrepancy. While that study used repeated presentations of brief ‘pips’ as the CS+ and CS−, continuous tones were used for these cues in our study. It is possible that theta oscillations in mPFC subserve different functions under these two conditions. Cue meaning might be signalled by theta activation elicited by repeated brief pips, whereas theta activity might better represent the resulting behavioural response to presentation of continuous cues^48^. Therefore mPFC theta activation during CS− presentation in the present study may signal safety and suppress inappropriate fear expression by coordinating communication between locally recruited neural ensembles^32^ and downstream areas of the neural circuitry underlying fear discrimination. Recent evidence demonstrating the entrainment of basolateral amygdala firing by mPFC theta oscillations during CS− presentation with successful fear discrimination lends support to this idea^34^. Importantly, we found a lack of mPFC theta activation during CS− presentation in females, suggesting that fear generalization involved reduced safety signalling and a corresponding lack of suppression of fear responding to the CS−. It is worth noting that we also found much lower basal theta activity in PL and IL in females, compared to males, before cue presentations at retrieval. Therefore this sex difference in basal theta activity in mPFC may also contribute to fear generalization and reduced safety signalling in females.

## Conclusion

This study confirms our previous findings showing that extended auditory fear discrimination training results in fear discrimination in males and fear generalization in females, based on cue-induced freezing at retrieval. It also builds on our previous results by showing that these sex differences in fear discrimination involve altered basal mPFC oscillations at retrieval. Males, but not females, also showed mPFC theta activation during CS− presentation and IL gamma activation during CS+ presentation at retrieval. To our knowledge this study is the first to characterize the neural circuitry involved in sex differences in fear discrimination by examining *in vivo* activity during retrieval testing. This study adds to growing evidence indicating a role for mPFC in mediating sex differences in learned fear inhibition more widely. From an evolutionary perspective, fear generalization might be adaptive under certain circumstances by promoting defensive behavior in response to a wider range of cues that are potentially predictive of threat. However, expressing fear inappropriately in response to harmless stimuli is maladaptive and may enhance the vulnerability to develop anxiety-related disorders such as PTSD^16,17^. These disorders are characterized by impaired inhibition of learned fear, are associated with mPFC dysfunction, and are more prevalent in women than in men, therefore these results may also have important translational relevance. Future studies examining the potential neuronal^33^, neural circuit^31,34,35,49–51^, and gonadal hormone^52^ mechanisms underlying sex differences in fear discrimination, both over the course of extended training and during later retrieval testing, may lead to a better understanding of why women are so much more vulnerable to developing anxiety-related disorders than men.

## Methods

### Animals

Young adult male and age-matched naturally cycling female Lister hooded rats (Charles River, UK) were used in this study. Rats weighed 200–325 g at the time of surgery and were housed 2–4/cage by sex in individually ventilated cages on a 12 hr light/dark cycle (lights on at 8:00), with free access to food and water. All experimental procedures were conducted with ethical approval from the University of Nottingham’s Animal Welfare and Ethical Review Body and in accordance with the Animals (Scientific Procedures) Act 1986, UK (PPL 30/3230).

### Surgery

Rats were administered pre-operative analgesic (buprenorphine) and isoflurane was used for anesthesia to ensure complete inhibition of the hindpaw withdrawal reflex during surgery. Body temperature was maintained ~37 °C using a homeothermic heating blanket (Harvard Apparatus Ltd, UK). Rats were placed in a stereotaxic frame (World Precision Instruments, UK) and the incisor bar was adjusted to keep the skull horizontal. An incision was made in the scalp and 4–8 jewellers screws were inserted into the skull. A small hole was drilled over the right mPFC and an eight-wire multi-electrode array (50 μm diameter Teflon-coated stainless-steel wires, with four wires 1 mm longer than the other four; NB Labs, US) was lowered into PL and IL (2.5 mm anterior and 0.5 mm lateral to bregma, 3.1 mm (PL) and 4.1 mm (IL) ventral to the brain surface). The electrode array was secured to the screws using dental cement. Sterile saline and analgesic (meloxicam) were administered at the end of surgery. Rats also received post-operative analgesic (buprenorphine and meloxicam) for two days after surgery and were allowed to recover 10–14 days before undergoing behavioural testing.

### Auditory fear discrimination paradigm

The apparatus (Med Associates, USA) used has been described in detail elsewhere^53^ and the auditory fear discrimination paradigm used was adapted from our previous study^26^. On Day 1, rats were habituated to two contexts (A and B) in which they received two presentations each of 2 and 9 kHz tones (30 s, 80 dB, 2 min inter-trial interval (ITI). Contexts A and B consisted of distinct visual (black and white stripes or spots on two walls), olfactory (40% ethanol or 40% methanol), and tactile (metal floor bars or white Perspex floor) cues. On Days 2–4 rats underwent once-daily sessions of discrimination training in context A, which consisted of five pairings of one tone (CS+; 30 s, 80 dB, 2 min ITI) with footshock (0.5 s, 0.5 mA, ending at tone offset) and five presentations of the other tone alone (CS−; 30 s, 80 dB, 2 min ITI)), with each CS+/US pairing being followed by a CS− presentation. The tones used for the CS+ and CS− (2 and 9 kHz tones as above) were counterbalanced between rats. On Day 5 rats received five presentations each of the CS+ and CS− alone in context B to assess discrimination retrieval (Fig. 1A). Each cue was followed by presentation of the other cue and the order in which the CS+ and CS− were first presented was counterbalanced between rats. Behaviour was recorded using a digital camera for later data analysis. Tone and footshock presentations were controlled automatically by a PC running MED-PC IV software (Med Associates, USA).

### Local field potential recordings

LFPs were recorded during retrieval by connecting the electrode array via a headstage, cable, commutator, and preamplifier to a Plexon Recorder system (Plexon Inc, US). LFPs were band-pass filtered at 0.7–170 Hz and digitized at 1.25 kHz. A cable connecting the Med Associates and Plexon Recorder systems was used to record events triggered by the MED-PC IV software to indicate the start of the retrieval session and the onset of each CS+ and CS− in the LFP data recording file.

### Histological verification of electrode placements

After completion of the experiments the rats were deeply anesthetized with sodium pentobarbital. Current was passed through random pairs of the electrode wires in PL and IL using an electrical stimulator (Grass Technologies, US) to deposit ferric ions at the electrode tip sites. Rats were transcardially perfused with 0.9% saline followed by a solution of 4% paraformaldehyde / 4% potassium ferrocyanide to mark the recording sites via the Prussian blue reaction. Brains were removed and stored in this solution until sliced into sections (80–200 µm) using a vibratome or microtome. Slices were stained for acetylcholinesterase to aid visualization of the electrode tip placements (Fig. 2a).

### Behavioural data analysis

Contextual fear before discrimination retrieval testing was inferred by measuring baseline freezing in the 2 min period before tones were presented at retrieval and was scored manually as described previously^26^. Sex differences in contextual fear were analyzed using an unpaired t-test. Discrimination at retrieval was inferred by measuring freezing in response to the CS+ vs CS− and was scored manually as described previously^26^. The mean percentage of freezing during each of the five CS+ and five CS− tones was calculated and used in the statistical analysis. Sex differences in freezing between CS+ and CS− presentation were analyzed using a two-way ANOVA, with sex and CS as between- and within-subject factors, respectively. The mean percentage of freezing during the CS+ and CS− tones with baseline freezing subtracted was also calculated and sex differences in this measure were analyzed in a separate analysis using a two-way ANOVA as above. Post-hoc comparisons were conducted using the Bonferonni’s test. The difference score was calculated by subtracting the mean percentage of freezing during CS− presentation from the mean percentage of freezing during CS+ presentation. Sex differences in the difference score were analyzed using an unpaired t-test. Freezing data is presented as the mean + SEM (or mean +/- SEM for the data presented in Fig. 3a,b) and the level of significance for the statistical comparisons was set at P < 0.05.

### Electrophysiological data analysis

LFP data from one representative channel in PL and IL was selected for analysis from each rat, based on minimizing electrical noise artefacts, and high-pass filtered at 4 Hz. LFP activity was examined visually and recordings with obvious electrical noise artefacts were omitted from the analysis. Spectral estimates for LFP data in each area before and during cue presentations in males and females were generated. Sex differences in basal theta (4–12 Hz) and gamma (30–45 Hz) power in PL and IL were first determined. Frequency analysis with multi-taper spectral estimates^54^ was conducted as previously described using custom Matlab scripts^55^. Multi-taper spectral estimates were constructed by splitting the 2 min basal period into 144 segments and averaging over *K* multi-taper windows, where *K* was used to tune the spectral bandwidth to theta (*K* = 5) or low gamma (*K* = 11) frequencies. Sex differences in basal theta and gamma power in PL and IL were determined using a log ratio test^56^. This ratio of variance test follows an F-distribution and a significance level is set to quantify differences in LFP power at specific frequencies. The log ratio test was applied as two-tailed to allow for both significant increases and decreases in theta or gamma power to be quantified.

Differences in theta and gamma power in PL and IL during CS+ vs CS− presentation in males and females were determined using time-frequency analysis with Morse wavelets^57^ and frequency analysis with multi-taper spectral estimates (see above). Fine detail in the LFP signals in PL and IL throughout each frequency band and cue duration was represented using wavelet periodograms constructed by averaging across trials (CS+ or CS−) and animals (males or females). Morse wavelet parameters were chosen to facilitate visual inspection of wavelet spectra in the two frequency bands of interest. This allowed for a qualitative analysis of changes in power throughout the cue duration. Multi-taper spectral analysis was used to quantify differences in theta and gamma power in each area between the CS+ and CS− in males or females after normalizing to basal theta and gamma power. Multi-taper estimates were constructed by splitting each 30 s cue into 36 segments and averaging over *K* multi-taper windows as above. Differences in theta and gamma power in PL and IL between CS+ and CS− presentation were determined separately for males and females using the log ratio test as above. LFP power data is presented as the mean + SEM and the level of significance for the statistical comparisons was set at P < 0.05.
